# Role of left atrial hypertension in pulmonary hypertension associated with bronchopulmonary dysplasia

**DOI:** 10.3389/fped.2022.1012136

**Published:** 2022-09-20

**Authors:** Rachel T. Sullivan, Megha D. Tandel, Shazia Bhombal, Gregory T. Adamson, Derek B. Boothroyd, Michael Tracy, Amanda Moy, Rachel K. Hopper

**Affiliations:** ^1^Division of Pediatric Cardiology, Department of Pediatrics, Vanderbilt University Medical Center, Nashville, TN, United States; ^2^Quantitative Sciences Unit, Department of Medicine, School of Medicine, Stanford University, Palo Alto, CA, United States; ^3^Division of Neonatal and Developmental Medicine, Department of Pediatrics, School of Medicine, Stanford University, Palo Alto, CA, United States; ^4^Division of Pediatric Cardiology, Department of Pediatrics, School of Medicine, Stanford University, Palo Alto, CA, United States; ^5^Division of Pulmonary Medicine, Department of Pediatrics, School of Medicine, Stanford University, Palo Alto, CA, United States

**Keywords:** left atrial hypertension, pulmonary hypertension, bronchopulmonary dysplasia (BPD), prematurity, diastolic dysfunction

## Abstract

Left atrial hypertension (LAH) may contribute to pulmonary hypertension (PH) in premature infants with bronchopulmonary dysplasia (BPD). Primary causes of LAH in infants with BPD include left ventricular diastolic dysfunction or hemodynamically significant left to right shunt. The incidence of LAH, which is definitively diagnosed by cardiac catheterization, and its contribution to PH is unknown in patients with BPD-PH. We report the prevalence of LAH in an institutional cohort with BPD-PH with careful examination of hemodynamic contributors and impact on patient outcomes. This single-center, retrospective cohort study examined children <2 years of age with BPD-PH who underwent cardiac catheterization at Lucile Packard Children's Hospital Stanford. Patients with unrepaired simple shunt congenital heart disease (CHD) and pulmonary vein stenosis (only 1 or 2 vessel disease) were included. Patients with complex CHD were excluded. From April 2010 to December 2021, 34 patients with BPD-PH underwent cardiac catheterization. We define LAH as pulmonary capillary wedge pressure (PCWP) or left atrial pressure (LAP) of at least 10 mmHg. In this cohort, median PCWP was 8 mmHg, with LAH present in 32% (*n* = 11) of the total cohort. A majority (88%, *n* = 30) of the cohort had severe BPD. Most patients had some form of underlying CHD and/or pulmonary vein stenosis: 62% (*n* = 21) with an atrial septal defect or patent foramen ovale, 62% (*n* = 21) with patent ductus arteriosus, 12% (*n* = 4) with ventricular septal defect, and 12% (*n* = 4) with pulmonary vein stenosis. Using an unadjusted logistic regression model, baseline requirement for positive pressure ventilation at time of cardiac catheterization was associated with increased risk for LAH (odds ratio 8.44, 95% CI 1.46–48.85, *p* = 0.02). Small for gestational age birthweight, sildenafil use, and CHD were not associated with increased risk for LAH. LAH was associated with increased risk for the composite outcome of tracheostomy and/or death, with a hazard ratio of 6.32 (95% CI 1.72, 22.96; *p* = 0.005). While the etiology of BPD-PH is multifactorial, LAH is associated with PH in some cases and may play a role in clinical management and patient outcomes.

## Introduction

Premature infants with bronchopulmonary dysplasia (BPD), or chronic lung disease of prematurity, are at risk for developing pulmonary vascular disease and associated pulmonary hypertension (PH). PH is defined hemodynamically by a mean pulmonary artery pressure (PAP) >20 mmHg (1). Approximately 20% of infants with BPD develop PH, and this risk increases with BPD severity (2–6). Immature alveolar and pulmonary vascular development, in combination with complex postnatal factors, results in decreased pulmonary vascular surface area, altered vascular tone, and pathologic vascular changes that culminate in PH (7–9). While lung disease is a main driver of pulmonary vascular disease in premature infants, there is increasing concern about the potential contribution of left atrial hypertension (LAH), which may result from left ventricular diastolic dysfunction or a volume load from a hemodynamically significant left to right shunt (10). LAH may have important clinical implications in the management of BPD-PH, as the use of pulmonary vasodilator therapy in the setting of unrecognized LAH may promote pulmonary edema and result in worsened respiratory status and/or prolonged need for respiratory support. Mourani et al. reported two cases of infants with BPD-PH in whom LAH was present at cardiac catheterization and thought to contribute to both pulmonary hypertension and significant diuretic-dependent pulmonary edema (11).

The incidence of LAH, which is definitively diagnosed by cardiac catheterization, and its contribution to BPD-PH is unknown. Some groups have explored the ability to identify diastolic dysfunction non-invasively by echocardiogram in premature infants (12, 13). However, differences in neonatal myocardial compliance and heart rate make these measures challenging to reliably obtain and interpret. Further, these echocardiographic measures lack the ability to quantitate left atrial pressure. Small, single-center reports of catheterization hemodynamics in BPD-PH suggest that the median left atrial pressure is normal at 7–10 mmHg (14–17). No studies have explored the impact of LAH on outcomes or sought to explore risk factors for the development of LAH. We hypothesize that a subset of infants with BPD-PH have concomitant LAH at cardiac catheterization, which may impact clinical outcomes.

## Methods

This retrospective cohort study evaluated patients with history of BPD-PH who underwent cardiac catheterization at Lucile Packard Children's Hospital Stanford from April 2010 to December 2021. Patients were included based on the following criteria: catheterization performed at our center, age <2 years at catheterization, PH as defined by criteria of mean PAP >20 mmHg, and diagnosis of bronchopulmonary dysplasia [based on gestational age and respiratory support at 36 weeks postmenstrual age (PMA)] (18). Infants with unrepaired simple shunt congenital heart disease [atrial septal defect (ASD), ventricular septal defect (VSD), and patent ductus arteriosus (PDA)] as well as pulmonary vein stenosis involving 2 or fewer veins were included. Patients with complex congenital heart disease, those who required cardiothoracic surgery, and those who had catheterization performed at an outside institution were excluded from this analysis. LAH was defined as a directly measured left atrial pressure (LAP) or pulmonary capillary wedge pressure (PCWP) ≥10 mmHg. In the presence of pulmonary vein stenosis, either direct LAP or PCWP from a lung segment with unobstructed pulmonary venous return was used. Acute vasoreactivity testing (AVT) was performed in a subset of patients. Hemodynamics were recorded at baseline and with maximal vasodilator therapy, which included 100% oxygen and/or 20 ppm inhaled nitric oxide, per interventional cardiologist discretion. Positive AVT testing was based on the Barst pediatric criteria of a decrease in mean PAP by at least 20% and an unchanged or increased cardiac index (19). Not all patients had arterial access during catheterization for accurate systemic vascular resistance to be calculated, so the Barst criteria of unchanged or decreased pulmonary to systemic vascular resistance ratio was unable to be utilized for AVT responsiveness in our cohort.

The primary aim of this study was to describe hemodynamics of an institutional cohort of patients with BPD-PH to determine incidence of LAH in this population. In this small pilot study, we explore the risk factors for LAH and examine the association of LAH with the composite outcome of tracheostomy and/or death. This study was approved by the Stanford University Institutional Review Board (IRB# 60898).

### Statistical analysis

Variables were compared across LAH status using descriptive statistics. Results were presented in counts and percentages for categorical variables and median [interquartile range (IQR)] for continuous variables. Absolute standardized differences (*d*) were also reported, which measured the effect size for the comparison of the two groups. Standardized differences measure the absolute difference between two groups relative to their internal variation and are calculated for continuous and binary variables. For variables with more than two categories, standardized differences are calculated using Mahalanobis distance (20). Differences >0.2 may be considered small, 0.5 medium, and 0.8 large. A Cox regression model was used to determine if LAH is associated with the risk for the composite outcome of tracheostomy and/or death. Hazard ratio estimates and 95% confidence intervals were calculated. Univariate logistic regression models were conducted on an a priori set of clinically relevant variables to identify risk factors for LAH. Odds ratios and 95% confidence intervals were reported. Multivariable models were not considered because of the small number of patients with LAH. All analyses were performed using SAS 9.4 (Cary, NC).

## Results

Between April 2010 and December 2021, 34 infants with BPD-PH underwent hemodynamic cardiac catheterization. Patient demographics and clinical characteristics are outlined in Table 1. There was a male predominance (65%) in this primarily non-Hispanic (74%) cohort. The median gestational age was 26.1 weeks PMA (IQR 24.9, 27.9 weeks PMA). Patients had median birth weight of 715 g (IQR 600, 895 g), with 35% (*n* = 12) meeting criteria for small for gestational age. A majority (*n* = 30, 88%) of the cohort had severe BPD. Congenital heart disease or pulmonary vein stenosis was present in 88% (*n* = 30) of the cohort, primarily with a PDA, ASD, or patent foramen ovale. Left ventricular systolic function was normal in all patients, and right ventricular systolic function was normal in 82% (*n* = 28) of patients, with the remainder having mild or moderate right ventricular systolic dysfunction. Patients were followed through April 1, 2022. Tracheostomy was performed in 32% (*n* = 11) of patients, and 18% (*n* = 6) of patients died (Table 1).

**Table 1:** Patient demographics and clinical characteristics (*N* = 34).

	**Total** ***N* = 34** ***n* (%)**	**With left atrial hypertension** **(≥10 mmHg)** ***n* = 11** ***n* (%)**	**Without left atrial hypertension** **(<10 mmHg)** ***n* = 23** ***n* (%)**	**Absolute standardized difference** ***(d)***
**Sex**				0.31
Male	22 (65)	6 (55)	16 (70)	
Female	12 (35)	5 (45)	7 (30)	
**Race**				0.79
Asian	10 (29)	2 (18)	8 (35)	
Pacific Islander	2 (6)	0 (0)	2 (9)	
Black	1 (3)	0 (0)	1 (4)	
White	10 (29)	5 (45)	5 (22)	
Other	11 (32)	4 (36)	7 (30)	
**Ethnicity**				0.33
Hispanic	9 (26)	4 (36)	5 (22)	
Non-Hispanic	25 (74)	7 (64)	18 (78)	
**Gestational age (weeks), median (IQR)**	26.1 (24.9, 27.9)	25.6 (24.9, 28.0)	26.6 (24.9, 27.7)	0.03
**Birthweight (g), median (IQR)**	715 (600, 895)	700 (420, 960)	740 (630, 895)	0.29
**Birthweight (percentile), median (IQR)**	27 (7, 56)	8 (1, 56)	39 (9, 57)	0.41
**SGA**	12 (35)	6 (55)	6 (26)	0.61
**Multiple congenital anomalies**	7 (21)	3 (27)	4 (17)	0.61
**BPD severity**				0.48
Mild	2 (6)	1 (9)	1 (4)	
Moderate	2 (6)	0 (0)	2 (9)	
Severe	30 (88)	10 (91)	20 (87)	
**Any type of CHD**	30 (88)	9 (82)	21 (91)	0.28
**PDA**	21 (62)	7 (64)	14 (61)	0.06
**ASD/PFO**	21 (62)	7 (64)	14 (61)	0.06
**VSD**	4 (12)	1 (9)	3 (13)	0.13
**PVS**	4 (12)	2 (18)	2 (9)	0.28
**Other**	3 (9)	0 (0)	3 (13)	0.55
**Echo LV systolic function**				—
Normal	34 (100)	11 (100)	23 (100)	
Mildly depressed	—	—	—	
Moderately depressed	—	—	—	
**Echo RV systolic function**				0.55
Normal	28 (82)	10 (91)	18 (78)	
Mildly depressed	3 (9)	1 (9)	2 (9)	
Moderately depressed	3 (9)	0 (0)	3 (13)	
**History of steroid use (*****N*** **=** **33)**	18 (55)	7 (64)	11 (50)	0.28
**Tracheostomy**	11 (32)	6 (55)	5 (22)	0.72
**Death**	6 (18)	4 (36)	2 (9)	0.70

IQR, interquartile range; SGA, small for gestational age; BPD, bronchopulmonary dysplasia; CHD, congenital heart disease; PDA, patent ductus arteriosus; ASD, atrial septal defect; PFO, patent foramen ovale; VSD, ventricular septal defect; PVS, pulmonary vein stenosis; LV, left ventricular; RV, right ventricular.

Clinical characteristics at time of catheterization and invasive hemodynamics are outlined in Table 2. Patients underwent catheterization at a median 3.4 months of age (IQR 3.1, 4.4 months), corresponding to a median 51.6 weeks PMA (IQR 42.3, 59.6 weeks PMA). Half of the cohort was supported at baseline with either invasive or non-invasive positive pressure ventilation (PPV) in the period preceding catheterization, which included infants requiring chronic intubation with mechanical ventilation, neonatal nasal intermittent positive pressure ventilation, and continuous or bilevel positive airway pressure. A majority (85%; *n* = 29) of infants were receiving scheduled diuretic therapy in the period preceding catheterization. Fifty-six percent of patients (*n* = 19) were on pulmonary vasodilator therapy at time of catheterization, most commonly with sildenafil in 47% (*n* = 16) of the total cohort. Baseline hemodynamics in our cohort demonstrated generally mild to moderate pulmonary vascular disease with a median mean PAP of 30 mmHg (IQR 27, 38 mmHg) and median indexed pulmonary vascular resistance (PVRi) 4.2 WU^*^m^2^ (IQR 3.2, 7.3 WU^*^m^2^). Median PCWP/LAP was 8 mmHg (IQR 8, 10 mmHg). Fifty percent (*n* = 17) of patients underwent intervention during catheterization: 14 patients underwent PDA device closure (2 of which were unsuccessful due to technical factors) and 3 underwent pulmonary vein balloon angioplasty. Hemodynamics utilized in analyses were obtained prior to intervention.

**Table 2:** Clinical characteristics at time of catheterization and invasive hemodynamics (*N* = 34).

	**Total** ***N* = 34** ***n* (%)**	**With left atrial hypertension** **(≥10 mmHg)** ***n* = 11** ***n* (%)**	**Without left atrial hypertension** **(<10 mmHg)** ***n* = 23** ***n* (%)**	**Absolute standardized difference** ***(d)***
Age at catheterization (months), median (IQR)	3.4 (3.1, 4.4)	3.4 (3.2, 4.5)	3.4 (2.9, 4.4)	0.14
Corrected gestational age at catheterization (weeks PMA), median (IQR)	51.6 (42.3, 59.6)	53.4 (42.7, 58.3)	48.0 (42.1, 60.1)	0.16
PPV preceding catheterization	17 (50)	9 (82)	8 (35)	1.09
Scheduled diuretic preceding catheterization	29 (85)	11 (100)	18 (62)	0.75
Pulmonary hypertension medications preceding catheterization	19 (56)	8 (73)	11 (47)	0.53
Inhaled Nitric Oxide	3 (9)	1 (9)	2 (9)	0.51
Sildenafil	16 (47)	7 (64)	9 (39)	0.87
Bosentan	3 (9)	3 (27)	0 (0)	0.67
Treprostinil	2 (6)	2 (18)	0 (0)	0
Tadalafil	1 (3)	1 (9)	0 (0)	0.45
**Baseline catheterization data**				
Respiratory support–RA	31 (91)	10 (91)	21 (91)	0.01
Respiratory support–Oxygen >50%	2 (6)	1 (9)	1 (4)	0.19
Respiratory support–iNO	2 (6)	1 (9)	1 (4)	0.19
Systolic PAP (mmHg), median (IQR)	45 (38, 53)	42 (35, 57)	45 (40, 52)	0.01
Diastolic PAP (mmHg), median (IQR)	22 (17, 26)	23 (18, 29)	22 (15, 26)	0.45
Mean PAP (mmHg), median (IQR)	30 (27, 38)	32 (27, 44)	30 (24, 38)	0.27
LAP/PCWP (mmHg), median (IQR)	8 (8, 10)	11 (10, 12)	8 (7, 8)	3.43
Qp:Qs, median (IQR)	1.4 (1.0, 1.9)	1.3 (1.0, 2.0)	1.4 (1.0, 1.9)	0.27
CI (L/min/m^2^), median (IQR)	3.4 (3.1, 4.4)	3.4 (3.2, 4.5)	3.4 (2.9, 4.4)	0.05
PVRi (WU*m^2^), median (IQR)	4.2 (3.2, 7.3)	3.5 (2.9, 8.2)	4.3 (3.5, 7.3)	0.08
Acute vasodilator testing performed	16 (47)	6 (55)	10 (43)	0.22
Vaso-reactive (*N* = 16)	6 (38)	1 (17)	5 (50)	0.76
**Intervention at catheterization**	17 (50)	7 (64)	10 (43)	0.48
PDA device closure	14 (82)	5 (71)	9 (90)	
Pulmonary vein balloon angioplasty	3 (18)	2 (29)	1 (10)	

IQR, interquartile range; PMA, postmenstrual age; PPV, positive pressure ventilation; RA, room air; iNO, inhaled nitric oxide; PAP, pulmonary arterial pressure; LAP, left atrial pressure; PCWP, pulmonary capillary wedge pressure; Qp:Qs, pulmonary to systemic flow ratio; CI, cardiac index; PVRi, indexed pulmonary vascular resistance; PDA, patent ductus arteriosus.

LAH was present in 32% (*n* = 11) of the cohort. Aside from PCWP/LAP, hemodynamics did not differ significantly between those with and without LAH. Patients with LAH were more likely to be small for gestational age (SGA), with 55% meeting SGA criteria and median birth weight at the 8^th^ percentile (IQR 1, 56 percentile) compared to the non-LAH cohort, which had 26% SGA with median birth weight at the 39^th^ percentile (IQR 9, 57 percentile, *d* = 0.61). Patients with LAH were more frequently supported at baseline with PPV in the period preceding catheterization (82%; *n* = 9) compared to the non-LAH cohort (35%; *n* = 8, *d* = 1.09). Patients with LAH were more likely to receive scheduled diuretic therapy prior to catheterization (100%; *n* = 11) compared to the non-LAH cohort (62%; *n* = 18, *d* = 0.75). Patients with LAH were also more likely to be on pulmonary vasodilator therapy, in particular sildenafil, prior to catheterization, with 64% (*n* = 7) of patients with LAH receiving sildenafil compared to 39% (*n* = 9) in the non-LAH cohort (*d* = 0.87). Both tracheostomy and death were more commonly encountered in the LAH cohort (*d* = 0.72 and 0.70, respectively).

**Figure 1:**
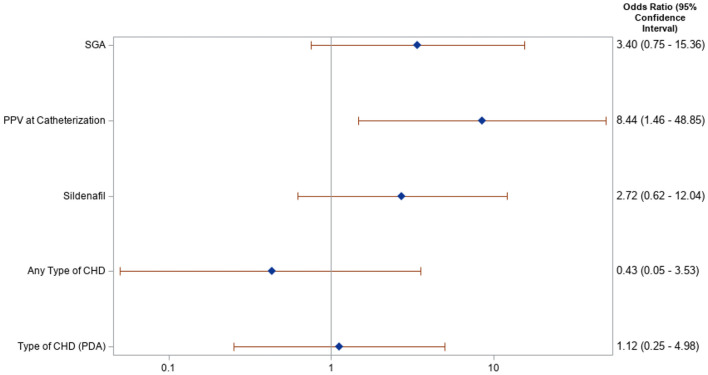
Forest plot of risk factors for left atrial hypertension: Risk for left atrial hypertension for select clinical factors is demonstrated in this Forest plot, along with associated odds ratio and p-value. Positive pressure ventilation (PPV) was the only variable that demonstrated significant risk for left atrial hypertension. SGA, small for gestational age; PPV, positive pressure ventilation; CHD, congenital heart disease; PDA, patent ductus arteriosus.

Univariate logistic regression was performed on a priori selected clinically relevant variables to evaluate for risk of development of LAH, shown in Figure 1. Requirement of baseline invasive or non-invasive PPV preceding catheterization was associated with increased risk for LAH with an odds ratio (OR) of 8.44 (95% CI 1.46, 48.85). SGA status, pre-catheterization use of sildenafil, and presence of underlying congenital heart disease (including specifically the presence of a PDA) were not significantly associated with increased risk for LAH. The presence of LAH was associated with increased risk for the composite outcome of tracheostomy and/or death, with a hazard ratio (HR) of 6.32 (95% CI 1.72, 22.96, *p* = 0.005).

**Figure 2:**
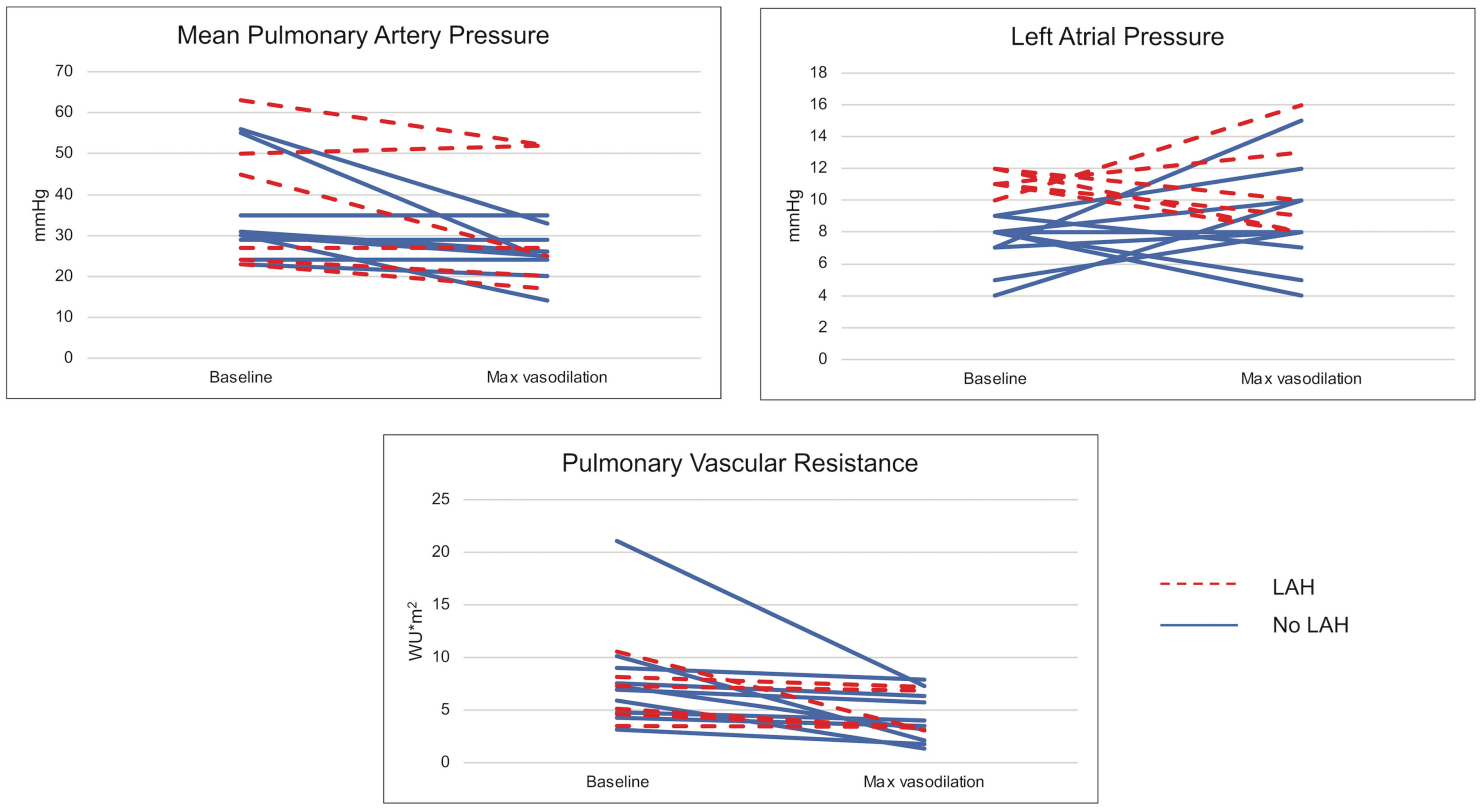
Hemodynamic changes with acute vasodilator testing. Changes in mean pulmonary artery pressure, left atrial pressure, and pulmonary vascular resistance are demonstrated at baseline and with maximal vasodilation (100% FiO2 ± inhaled nitric oxide) utilized in acute vasodilator testing, which was performed in 16 patients.

Acute vasodilator testing (AVT) was performed in 47% of patients (*n* = 16), including 55% (*n* = 6) of those with LAH and 43% (*n* = 10) of those without LAH. Hemodynamic response to AVT was variable, as depicted in Figure 2. Fewer patients with LAH responded positively to AVT (17%) compared to the non-LAH cohort (50%) (*d* = 0.76). While not all met criteria for positive AVT, some patients with LAH showed improvement in both mPAP and LAP with AVT. Conversely, a subset of those without LAH at baseline developed LAH with AVT (Figure 2).

## Discussion

This retrospective cohort study is the first to describe the incidence of LAH in patients with BPD-PH. We found LAH in nearly one-third of infants whose hemodynamics have been evaluated by cardiac catheterization. The need for baseline PPV preceding catheterization was associated with increased risk for LAH. LAH was associated with increased risk of tracheostomy and/or death.

The pulmonary vascular changes associated with BPD that lead to PH are well-described (7–9). However, LAH as a potential contributor to the cardiopulmonary status of a premature infant is poorly understood. Premature birth alters myocardial structure, with cardiomyocyte hypertrophy and increased collagen deposition (21). This may predispose to altered diastolic function, with potential for resultant LAH. Cardiac shunt lesions, particularly a persistent PDA, are common in premature infants (22). With a hemodynamically significant PDA, left heart volume overload can also cause clinically relevant LAH, particularly in preterm infants who may be predisposed to diastolic dysfunction. Impaired diastolic function has been demonstrated by echocardiographic studies, with hemodynamically significant PDA being a potential contributor to these indices (12, 13, 23). However, these remain indirect markers of diastolic dysfunction, and there is some evidence that brings into question the reliability of these markers in patients with pulmonary hypertension (24).

Therefore, we examined directly measured PCWP/LAP in the catheterization lab. One limiting factor is the lack of definition for hemodynamic norms, particularly normal left atrial pressure, in premature infants. The formal cut-off for post-capillary pressure elevation is a PCWP of ≥ 15 mmHg according to the World Symposium on Pulmonary Hypertension classification schema (25). However, normal LV diastolic pressures in healthy children have been demonstrated to be significantly lower than this at a mean of 7.5 mmHg (standard deviation 2.2 mmHg) (26). Premature infants with BPD may develop clinically relevant symptoms at a lower LA pressure than 15 mmHg, with an initial case series highlighting clinically significant pulmonary edema related to diastolic dysfunction in premature infants with PCW pressures of 12 and 17 mmHg (11). For these reasons, our group used a cut-off of 10 mmHg to define LAH to be inclusive of patients with mild, but potentially clinically important, elevations of LA pressure.

The increased use of diuretics and positive pressure ventilation in patients with LAH suggests that even mild LAH may correlate with increased clinical symptoms, although causality cannot be attributed. Further, a majority of this cohort had underlying CHD with left to right shunt, which likely contributed to the benefit of diuretic therapy. Additionally, LAH was associated with increased risk for the composite outcome of death and/or tracheostomy. It is unclear whether worse lung function predisposes to LAH or if LAH worsens parenchymal lung disease by causing pulmonary edema that increases need for diuretics and ventilator support. Larger studies will be required to investigate this further.

Congenital heart disease was not associated with LAH in our cohort. We anticipated that the presence of a hemodynamically significant PDA would be a significant contributor to LAH. Contrary to our initial hypothesis, CHD, including PDA, was not associated with significantly increased risk for LAH by univariate analysis. This is particularly notable given that many of the PDAs present in our cohort were likely hemodynamically significant, with 41% undergoing attempted PDA device closure at time of catheterization. Additionally, pulmonary vasodilator use was not associated with worse LAH. These medications were frequently in use before catheterization, particularly as expert consensus suggests the initiation of pulmonary vasodilator monotherapy without catheterization is reasonable in uncomplicated BPD-PH when balancing risks associated with catheterization (27, 28). While the increased pulmonary blood flow that may result from pulmonary vasodilator therapy may pose theoretic risk for LAH in the less compliant myocardium of the premature infant, the use of sildenafil was not associated with increased risk for LAH in our cohort. Similarly, few patients showed significant worsening of LAH with AVT, although testing did unmask LAH in a few subjects.

Limitations of this study include its retrospective nature and small sample size in a single center. The small size and heterogeneous nature of the population precluded multivariate analysis. Selection bias may be present as we included only patients who underwent cardiac catheterization. At our center, infants with BPD-PH are often treated with diuretic therapy and pulmonary vasodilator monotherapy before consideration of cardiac catheterization, making our study potentially skew toward a sicker cohort in whom catheterization was required to guide clinical management. However, the relatively mild elevation of pulmonary artery pressure and pulmonary vascular resistance might suggest this is not the case. In this cohort of infants with BPD-PH, cardiac catheterization was performed under general anesthesia with patients intubated and mechanically ventilated with optimized respiratory mechanics. As such, the hemodynamics may reflect the “best case scenario” rather than the clinical baseline. Conversely, mechanical positive pressure ventilation in the catheterization lab may negatively impact bi-ventricular compliance and result in increased left atrial pressure compared to clinical baseline in those who who are not supported with chronic PPV (29). Interpretation of the hemodynamics (and response to AVT) may also be confounded by the fact that most patients were treated with pulmonary vasodilator therapy and diuretics at time of catheterization.

This small pilot study is the first to examine the incidence and clinical impact of LAH in patients with BPD-PH. Clinically, this study raises the question of whether some patients should undergo earlier hemodynamic evaluation with cardiac catheterization to assess for LAH. Based on this pilot data, one may consider low threshold for diuretic therapy in premature infants with PH. Earlier catheterization should be considered in patients with echocardiographic evidence of PH who have increasing diuretic requirements or inability to wean respiratory support, especially following initiation of pulmonary vasodilator therapy. Additional larger, multi-institutional studies are needed to further define the definition of LAH in this population and further investigate its clinical impact on BPD-PH.

## Data availability statement

The raw data supporting the conclusions of this article will be made available by the authors, without undue reservation.

## Ethics statement

The studies involving human participants were reviewed and approved by Stanford University Institutional Review Board. Written informed consent from the participants' legal guardian/next of kin was not required to participate in this study in accordance with the national legislation and the institutional requirements.

## Author contributions

RS contributed to project design, data collection/analysis, and was responsible for primary manuscript composition. MDT and DB performed statistical planning and analysis. RH contributed as senior author with primary role in project design, data analysis, and manuscript preparation. SB, GA, MT, and AM contributed specialty-specific input in project development and manuscript review in the fields of neonatology, interventional pediatric cardiology, pediatric pulmonology, and neonatology, respectively. All authors demonstrated significant academic contribution to justify authorship and were involved in manuscript review and final manuscript approval.

## Conflict of interest

The authors declare that the research was conducted in the absence of any commercial or financial relationships that could be construed as a potential conflict of interest.

## Publisher's note

All claims expressed in this article are solely those of the authors and do not necessarily represent those of their affiliated organizations, or those of the publisher, the editors and the reviewers. Any product that may be evaluated in this article, or claim that may be made by its manufacturer, is not guaranteed or endorsed by the publisher.
